# Preparing Physical and Occupational Therapists to Be Health Promotion Practitioners: A Call for Action

**DOI:** 10.3390/ijerph15020392

**Published:** 2018-02-24

**Authors:** David M. Morris, Gavin R. Jenkins

**Affiliations:** 1 Department of Physical Therapy, School of Health Professions, University of Alabama at Birmingham, 1716 9th Avenue South, Birmingham, AL 35294, USA; 2 UAB Department of Occupational Therapy, School of Health Professions, University of Alabama at Birmingham, 1716 9th Avenue South, Birmingham, AL 35294, USA; jenkinsg@uab.edu

**Keywords:** health promotion, wellness, education, physical therapy, occupational therapy

## Abstract

Experts around the world support the integration of health promotion and wellness (HPW) services into traditional health care services. If successfully executed, the addition of HPW services would reduce rates of death and disability and significantly reduce health care costs. While all health care providers should be engaged in providing HPW services, many believe that physical therapists (PTs) and occupational therapists (OTs) are uniquely positioned to provide these services. However, research suggests that clinicians in both fields may fall short in doing so. Likewise, research indicates that entry-level educational programs inadequately prepare PT and OT students to be HPW practitioners. The overall purpose of this paper is to provide recommendations to educators for preparing PT and OT students and clinicians to better meet the HPW needs of the clients and patients they serve.

## 1. Introduction

With escalating health care costs and changing demographics in the United States, the U.S. Department of Health and Human Services has emphasized health promotion and disease prevention to improve the life expectancy, health, and quality of life of all Americans [1]. Health promotion is a prevention strategy and the process of enabling people to increase control over, and improve, their overall health [2]. A key purpose of health promotion is to prevent disease and disability in individuals and populations. Although typically occupational therapists and physical therapists offer services focused on individuals or groups presenting with specific diseases or disabilities, more recently these models are being challenged. Within emerging systems that focus increasingly on health promotion, occupational therapists and physical therapists must shift their perspective and adapt to the prevailing philosophy of preventing or reducing the incidence of illness and noncommunicable diseases, accidents, injuries, and disabilities in the population.

The World Health Organization defines noncommunicable diseases (NCDs) as noninfectious and non-transmissible diseases that may be caused by genetic and behavioral factors and generally have slow progression and long duration [3]. Examples include cardiovascular diseases, cancer, chronic respiratory diseases, diabetes, stroke, and osteoporosis. NCDs are the leading cause of death and disability globally and account for 70% of deaths annually [4]. Health care costs associated with managing NCDs are staggering. It is estimated that, if not addressed adequately, the costs associated with managing NCDs will exceed $47 trillion between 2011 and 2030 [4]. 

It is widely accepted that occupational therapists (OTs) and physical therapists (PTs) could dramatically decrease the burden of disease and disability if they effectively integrated health promotion and wellness (HPW) philosophies into their practice [5,6,7]. Supporting healthy lives would mean facilitating a full range of functional capacities across the lifespan, allowing people to enter into satisfying relationships with others, to work, and to play. This ideal is consistent with the beliefs of both professions that engagement in functional activities supports health and leads to a productive and satisfying life. Research suggests, however, that OTs and PTs inadequately and inconsistently do so [8,9,10,11,12]. Evidence also indicates that PT and OT entry-level educational programs include health-promotion and disease-prevention learning objectives in their curricula [13]. However, important topics/content may be insufficiently addressed or omitted altogether. Also, it appears that most educational programs primarily address these issues theoretically and through didactic learning experiences, with little attention to or available opportunities directed at application and clinical experiences related to these topics. Of note, educators in other health care professions (e.g., nursing and medicine) have addressed this issue [14,15]. While professional duties for these groups may differ from those of PTs and OTs, there may be considerable overlap in educational content that supports health promotion and disease prevention.

The purposes of this paper are to (1) state the case for PTs and OTs as health-promotion practitioners, (2) identify essential entry-level curriculum content for preparing PT and OT students for this role, and (3) recommend educational strategies/practices to assure preparation. For the purposes of this paper, we are addressing the role of PT and OT professionals, but acknowledge that these practices, wholly or in part, relate to other health care providers. Also, while the emphasis of this paper is on entry-level education, we will also address strategies and competencies for enhancing HPW competencies for experienced rehabilitation therapists by way of post-professional education. Many of the views expressed in this paper come from professional standards and the opinions of experts. Some, however, come from opinions developed from our experience as PT and OT educators and administrators. Where appropriate, we will identify statements coming from our experience.

## 2. Role as Health Promotion Practitioners

Both occupational therapy (OT) and physical therapy (PT) services are provided to clients/patients of all age groups, infants through older adults, from a variety of socioeconomic, cultural, and ethnic backgrounds, who possess or who are at risk for impairments, activity limitations, or participation restrictions. Both professions recognize that health and well-being are supported when individuals are able to engage in activities that promote quality of life through a healthy lifestyle [16,17].

As a result, leaders, academics, and practitioners in PT and OT agree that both professional groups play an important role in HPW. According to the American Occupational Therapy Association’s (AOTA) *Occupational Therapy Practice Framework: Domain and Process* [16] and the American Physical Therapy Association’s (APTA) *Guide to Physical Therapist Practice* [17], HPW is an essential element in the practice of the respective profession. Examples of HPW activities are provided in the Guide to Physical Therapist Practice and are presented in Table 1 [17]. While written specifically for PT, we believe that OT professionals would likely engage in similar activities. The professional organizations for both groups have adopted positions supporting this role among all clinicians. In a 2016 position adopted by APTA, the PT’s role in HPW is described as a “dynamic link between health and health care.” [18]. A 2015 APTA position statement identifies health priorities in the areas of prevention, wellness, fitness, health promotion, and the management of disease and disability [19]. These priorities are listed in Table 2. AOTA asserts that health and well-being are intricately linked to the daily occupations that people choose to participate in, and that occupational therapy practitioners play an important role in promoting health and preventing disease and disability. Occupational therapy practitioners believe that “health is strongly influenced by [individuals] having choice and control in everyday occupations” and, as such, occupation is a determinant of health [20,21]. Similar positions have been taken by the World Federation of Occupational Therapists [22] and the World Confederation for Physical Therapy [23].

These position statements suggest that PTs and OTs are particularly well suited to provide HPW for different populations and challenges. Dean [6] states that PTs are particularly well positioned to provide health promotion services because of their (1) educational background, which spans pathology and pathophysiology in relation to anatomy and exercise; (2) expertise and skills in promoting exercise, fitness, and wellness; (3) opportunities for frequent and relatively lengthy contact with patients/clients; and (4) often close and trusting relationships with their patients/clients. Additionally, AOTA states that OTs’ “unique perspective helps clients adapt and organize their daily occupations or activities related to self-care, home management, community participation, education, work and/or leisure into daily routines to prevent and minimize dysfunction, promote and develop a healthy lifestyle, and facilitate adaptation and recovery from injury, disease, or developmental challenges” [21].

There are several critical roles for PT and OT practitioners in health promotion and disease or disability prevention: to promote healthy lifestyles, to emphasize physical exercise/fitness and activity/occupation as essential elements of health promotion strategies, and to provide interventions, not only for individuals but also for populations. 

## 3. Recommended Curricular Content

Decisions regarding adding content to an already “packed” curriculum should be approached judiciously, particularly when some accreditation standards put heavy emphasis on content that is to be covered in the respective PT and OT education programs. However, our experience as educators has led us to see the need to find the right balance between content and other skills needed for practice readiness, such as creativity and curiosity, two skills that employers are saying are vital for our graduates. Therefore, if the professions accept the importance of HPW within the shifting focus of health care, they need to explore ways to rebalance curricula, through either incremental or more radical change, to integrate HPW curricular content and skills development.

Recommendations regarding competencies needed to practice HPW have been provided. Also, this content is addressed in accreditation standards for both PT and OT education [24,25]. Below, we provide recommendations regarding topics that we believe are helpful in preparing rehabilitation therapists to be HPW practitioners. Our recommendations are informed by suggested competencies, accreditation standards, and our personal opinions. 

### 3.1. The Language of Health Promotion

To practice HPW, clinicians need an in-depth understanding of critical terms and concepts related to HPW. This is particularly important, as many of these concepts (e.g., health, wellness, fitness, occupational imbalance, deprivation, and alienation) are multidimensional and include aspects that are not always obvious to the lay public. Further, omitting some of these aspects could render the HPW efforts less effective or irrelevant. Table 3 includes particularly important concepts, definitions, and references that may guide educators [17,26,27,28,29,30,31]. To promote the application of these concepts, a myriad of both clinical and social examples should accompany theoretical discussions. 

### 3.2. Health Behavior Change Theory

While important, knowledge about healthy living is generally insufficient to effect health behavior change. Individuals change their health practices when they perceive a need to change, are ready to change, have the necessary knowledge, skills, and tools to change, and have a supportive environment [28]. The multidimensional nature of health behavior highlights the complexities of promoting health lifestyle behaviors. Occupation-based and physical therapy theories can be complemented with health behavior change theories to assist us in evaluating the factors that drive health behaviors and then develop behavior-based interventions to promote change when needed. Many relevant health behavior change theories exist; some of the more common theories used are the health belief model, the transtheoretical or stages of change theory, social cognitive theory, the theory of planned behavior, and precaution adoption theory [28,32]. Within these theories, common constructs can be found that are particularly important for promoting behavior change. Notable examples are the constructs of self-efficacy, readiness to change, choice, meaning, balance, satisfaction, opportunity, self-actualization, perceived barriers, and environmental influences. PTs and OTs are in a prime position to recognize these constructs and use theory to describe, explain, and predict behavior change in people and populations. The field of health behavior is growing, and many health behavior change theories could be included in a PT or OT educational curriculum. We recommend that students be introduced to the general concept of health behavior change theory and some of the more established theories, such as those mentioned above, be used to illustrate application to patients/clients. Further, there should be an emphasis on some of the more common constructs, such as those previously mentioned. 

### 3.3. Health Data Management

Different populations face different health issues and priorities. In order to anticipate and prepare for meeting these challenges, PTs and OTs must be aware of and skilled at using relevant health data. Health data surveillance systems are particularly helpful for this purpose. These systems are particularly useful, as they are longitudinal in nature (i.e., track data over time so that trends can be observed) and can usually be examined according to specific demographic characteristics (e.g., age, race, geographic location, gender) that PTs and OTs are interested in exploring. In this way, OTs and PTs can tailor their searches to meet the needs of the patients/clients they will most likely see in their practice setting. Important surveillance systems include the Behavioral Risk Factor Surveillance System, the National Health and Nutrition Examination Survey, the National Health Care Survey, the National Health Interview Survey, and the U.S. Census. All of these surveillance systems, except for the U.S. Census, can be accessed at the Centers for Disease Control and Prevention website: www.cdc.gov. U.S. Census data can be accessed at www.census.gov. 

### 3.4. Health Communications

Conversations regarding lifestyle behaviors and health can be quite sensitive. At a fundamental level, the holistic, system-oriented, and client-centered approaches of PTs and OTs support communication on these matters. In addition, specific communication strategies have been developed to promote understanding of, motivation for, and adherence to health advice to optimize health and wellness. Three communication strategies are commonly mentioned when discussing health-focused clinical care: motivational interviewing (MI), the “5 A’s,” and the “5 R’s” [33,34]. Miller and Rollnick [33] originally developed MI to address problem drinking. The counseling approach is described as nonjudgmental, nonconfrontational, and nonadversarial. PTs and OTs skilled at MI use four basic skills: asking open-ended questions, providing affirmations, listening in a reflective manner, and periodically providing summary statements. Using MI, therapists assist patients/clients with exploring and resolving their ambivalence toward their unhealthy lifestyle behaviors and developing potential strategies to improve them. MI is a relevant and usable tool in both PT and OT practice to promote partnership skills when working in health promotion and prevention settings.

The 5 A’s counseling approach was originally developed for smoking cessation. It has been used for a variety of health issues [34]. The approach is rooted in behavior change theory and can be used quickly and effectively in the clinical setting. The 5 A’s are most effective when the patient/client is already interested and at least somewhat motivated to make a change in behavior. The 5 R’s, however, represent a counseling approach that is designed for patients/clients who are resistant to changing their behavior [34]. Major principles underlying these three communication strategies are listed in Table 4. For more detailed descriptions, readers are encouraged to explore references [33,34].

### 3.5. Lifestyle Medicine/Health-Focused Care

Health-focused care (HFC) can be defined as integrating lifestyle practices into conventional medicine to lower the risk of chronic disease and, if disease is already present, to serve as an adjunct to therapy [6]. Lifestyle issues commonly addressed with HFC include physical activity, nutrition optimization, weight management, stress management, smoking cessation, sleep hygiene, and alcohol moderation. While recognizing the role of PT and OT in health promotion and disease and disability prevention, it is important to acknowledge and respect the contributions of other health care professionals. This type of intervention is the responsibility of all members of the health care team and should be provided routinely and systematically for all patients/clients. PTs and OTs can provide assessments for health risks, teach strategies to incorporate healthy habits and routines, educate about the importance of relaxation and rest, and provide other skills training to promote health and well-being in their patients’/clients’ lives. Faculty in the School of Health Professions at the University of Alabama at Birmingham (UAB) developed and validated a practice model for use of HFC in rehabilitation clinical practice. The UAB HFC model is useful for educational purposes, and is particularly useful for exploring clinical decision-making and application of HFC to patient/client cases [35]. Major steps in the model include (1) performing a needs analysis for HFC, (2) determining a patient’s/client’s need for lifestyle behavior change (LBC), (3) collaborating with patient/client about LBC needs, (4) providing LBC interventions, and (5) assessing LBC outcomes. More details about specific strategies can be found in reference [35]. 

### 3.6. Health Program Planning and Evaluation

Developing and executing health promotion and prevention programs can be taxing from the financial, time, and energy perspectives. As such, these programs should be carefully planned and evaluated. Program planning and evaluation models specific to health programs can be invaluable for this purpose. The PRECEDE-PROCEED model is useful for diagnosing health issues and developing highly relevant programs to address them [36]. The acronym stands for predisposing, reinforcing, and enabling constructs in educational/environmental diagnosis and evaluation (PRECEDE) and policy, regulatory, and organizational constructs in educational and environmental development (PROCEED). Developed by Green and Kreuter, the model is described as “beginning at the end”, as it first sets out to identify the quality-of-life concerns of the target audience, considered to be among the most important goals for any health program. The model calls on program planners to segment the target audience (i.e., identify the prevailing characteristics of the anticipated participants), as program elements must consider important participant demographic factors (e.g., age, gender, race, socioeconomic status). Green and Kreuter also promote the “principle of participation”, which supports involving target participants and other stakeholders in the planning, implementation, and evaluation processes. This is critically important, as these individuals are most likely to identify relevant program elements that will ultimately be successful at addressing health issues. The PRECEDE portion of the model is most helpful for diagnosing and developing relevant program elements. The PROCEED portion of the model is most helpful for implementing, refining, and evaluating the program. Major components of the model are listed and described in Table 5. Other program planning models are available (e.g., social marketing, RE-AIM (The Reach, Effectiveness, Adoption, Implementation and Maintenance (RE-AIM) framework)) and could also be useful in HPW educational efforts. Readers are encouraged to examine reference [36] for more details on the model.

### 3.7. Community Health Promotion and Advocacy

To be effective, health promotion efforts cannot focus only on intervention at the individual level. Because of the inextricable and reciprocal links between people and their environment, larger groups, organizations, communities, populations, and government policy-makers must also be considered for intervention [28]. Both OT and PT already acknowledge that clients/patients are typically classified as persons (including those involved in the care of individuals), groups (collectives of individuals, e.g., families, communities), and populations (collectives of groups of individuals living in a similar locale). Health-promotion services occur in various settings, including but not limited to hospitals, skilled nursing facilities, continuing care retirement centers, community organizations, schools, and workplaces.

Community health promotion can be defined as the application of a variety of methods for education and mobilization of community members in actions to resolve health issues and problems that affect the community. Examples of such programs include group processes, mass-media campaigns, strategic planning and skills training with community organizations, and advocacy initiatives related to legislation and policy-making. Critical differences exist between health programs implemented in clinical and community settings. Gahimer and Morris described some of these differences, including units of service, activity focus, boundaries of service, coordination, patient/client autonomy, and predictability of events [37].

A critical resource for exploring community health is the Healthy People Program [1]. Established in the 1990s, Healthy People provides consensus- and science-based 10-year objectives for improving the health of all Americans. By establishing benchmarks and monitoring progress toward them, the program sets priorities for health programs, encourages collaboration across communities and sectors, empowers individuals to make better health decisions, and measures the impact of prevention activities. In addition to providing details about the nation’s health priorities, the website includes health data, evidence-based resources, program planning resources, and access to webinars and other educational tools.

## 4. Other Important Concepts and Characteristics

In addition to addressing topics and concepts discussed in the previous section of this paper, other educational topics and strategies should be considered. These factors are not specific to HPW, yet are critical to assuring that these concepts are effectively integrated into PT and OT clinical practice. While these educational concepts and characteristics should be included throughout the curriculum, efforts should also be made to align them with HPW concepts. 

*Interdisciplinary collaboration* is essential in all aspects of PT and OT practice [38]. The multidimensional nature of HPW issues, and the need for multiple services to address them, makes interdisciplinary collaboration particularly important when preparing HPW practitioners. Also, depending on the lifestyle issue under consideration, a patient’s/client’s need for HPW services may go beyond the PT or OT scope of practice, requiring referral to and/or collaboration with other professionals. PT and OTs must therefore recognize when these situations occur, be aware of services that are available from other professional groups, and know how to collaborate with others within and outside of their profession to provide the HPW services needed. Doing this requires an understanding of roles and respect for other professionals, effective communication, and teamwork, all skills that can be, and should be, fostered in entry-level education. 

A related yet slightly different skill is consultation, the rendering of professional or expert opinion or advice. A consulting PT or OT applies highly specialized knowledge and skills to identify problems, recommend solutions, or produce specified outcomes or products in a given amount of time on behalf of patients/clients. At other times, the PT or OT seeks consultative services from another provider to inform the plan of care, or seeks services beyond the professional or personal scope of practice (shares responsibility for patients/clients). Consultation can be facilitated by establishing consultancy networks where multiple professional groups form a close working relationship to facilitate cross-referrals and collaboration. Entry-level PT and OT students should be exposed to successful models for establishing such networks.

Most PT and OT services are paid for, at least partially, by health insurance. Few HPW services are covered by health insurance policies [6]. Further, “lack of payment” has been identified by therapists as a significant barrier to providing HPW services to patients/clients [9,10,11,12]. If assisted with managing the barrier of lack of payment for HPW services, PTs and OTs would be more likely to provide these services. As such, PTs and OTs must consider *innovative business practices* for integrating HPW services into clinical practice. For example, cash-based services are feasible and profitable when used with many patients/clients. Federal, industry, and community grants may fund HPW programs that can support PT and OT services. Finally, PT and OT clinics can reduce the costs associated with providing HPW services by effectively using support personnel and integrating trustworthy and readily available HPW educational resources (e.g., MyPlate.gov, NCHPAD.org) into traditional PT and OT services. 

Bodner and colleagues [13] observed that while many health profession educational programs included HPW content, it was delivered primarily at a theoretical level, with few opportunities to apply the concepts clinically. This situation is further challenged by the fact that PT and OT clinical instructors may be insufficiently delivering HPW services in the clinic. Educational programs should look for creative ways to provide clinical application experience with HPW services. In addition to establishing affiliation agreements with traditional PT and OT clinics, community health settings should be considered for clinical education experience. Students assigned to traditional clinics could also be assigned projects specific to HPW issues. Finally, service learning projects could be added to HPW coursework to ensure application of concepts discussed. 

With regard to teaching formats, HPW content could be effectively delivered in a PT or OT curriculum in a variety of ways (e.g., entire course, integrated into other courses). Regardless, we recommend that these concepts are introduced early in the curriculum. Doing so will emphasize HPW as an essential role for PT and OT practice and will also allow for later coursework to include examples of HPW in clinical practice.

At least two arguments support the need to increase post-professional opportunities for developing skills with HPW: (1) research indicating that PT and OT professionals may currently be using these skills in practice inadequately and inconsistently [9,10,11,12], and (2) research suggesting that entry-level PT and OT educational programs have not been preparing graduates to provide HPW services [13]. AOTA and APTA have provided multiple resources to promote professional development in this area, including articles, continuing education courses, podcasts, handouts, bibliographies, and resource lists. We believe that post-professional education opportunities should emphasize the same content/topics recommended for preparing entry-level students to provide HPW. We also believe that if entry-level students are adequately prepared, enthusiastic about this role, and confident in their ability to carry out these skills, they will serve as critical “change agents” to integrate these skills in the clinics where they work. As such, another critical skill for preparing entry-level students as HPW practitioners is serving as advocates both within and outside of their respective professions.

## 5. Conclusions

Experts advocate for the evolution of U.S. health care from a system that provides mostly “sick care” (i.e., providing services to patient/clients only after they have developed disease and functional decline) to one that equally offers “health care” (i.e., providing disease prevention as part of routine clinical care). Incorporating HPW services into standard care is expected to dramatically reduce deaths and disease morbidity and significantly reduce health care costs. Achieving this goal will require a significant paradigm shift in current PT and OT clinical practice. Since it is believed that PT and OT educational programs in the United States are currently inadequately preparing graduates to be HPW practitioners, a similar paradigm shift should occur with regard to educational practices. AOTA and APTA have described and emphatically endorsed the role of OTs and PTs as HPW practitioners. Experts have also detailed competencies needed to fulfill these roles. This article was written to offer recommendations concerning HPW content and delivery characteristics to institutions/educators that provide entry-level and post-professional education for PTs and OTs. If achieved, the future of PT and OT clinical care will be enhanced significantly and better outcomes for patients/clients will be realized. 

## Figures and Tables

**Table 1:** Examples of prevention and health, wellness, and fitness promotion activities in which physical therapy (PT) and occupational therapy (OT) clinicians might engage.

Possible HPW Activities
• Workplace redesigns, back schools, strengthening, stretching, endurance exercise programs, and postural training to prevent and manage low back pain (primary)
• Ergonomic redesigns to prevent job-related disabilities, including trauma and repetitive stress injuries (primary)
• Exercise programs, including weight bearing and weight training, to increase bone mass and bone density (especially in older adults with osteopenia and osteoporosis) (secondary or tertiary, depending on nature of condition)
• Exercise programs, gait training, and balance and coordination activities to reduce the risk of falls—and the risk of fractures from falls—in older adults (primary)
• Exercise programs and instruction in activities of daily living and instrumental activities of daily living to decrease utilization of health care services and enhance function in individuals with cardiovascular/pulmonary disorders (secondary or tertiary, depending on nature of condition)
• Exercise programs, cardiovascular conditioning, postural training, and instruction in activities of daily living and instrumental activities of daily living to prevent disability and dysfunction in women who are pregnant (secondary)
• Broad-based consumer education and advocacy programs to prevent problems (e.g., prevent head injury by promoting the use of helmets) (primary)
• Exercise programs to prevent or reduce the development of sequelae in individuals with lifelong conditions (tertiary)
• Programs for healthy lifestyle for individuals to decrease the risk of noncommunicable disease and disability

**Table 2:** American Physical Therapy Association (APTA) position: health priorities for populations and individuals [16].

Category	Targets
Active Living	Aging individuals and populations Individuals and populations of all ages with health disparities Individuals and populations of all ages with chronic conditions, disabilities, and diseases that impact their ability to remain independent and physically active
Injury Prevention	Fall prevention Workplace injury prevention Community-based injury prevention
Secondary Prevention in Chronic Disease and Disability Management	Diseases and disabilities that impair an individual’s body function or structure Diseases and disabilities that limit an individual’s activity Diseases and disabilities that restrict an individual’s participation in society Diseases and disabilities that require modification of environmental factors to allow for full participation in society
Education, Behavioral Strategies, Patient Advocacy, Referral Opportunities, and Supportive Resources after Screening	Stress management Smoking cessation Sleep health Nutrition optimization Weight management Alcohol moderation and substance-free living Violence-free living Adherence to health care recommendations

**Table 3:** Critical health promotion and wellness (HPW) concepts and terms.

Concept/Term	Definition
Health [26,27]	State of being associated with freedom from disease and illness that also includes a positive component (wellness) that is associated with quality of life and positive well-being (APTA) Not merely absence of disease…a state of complete **physical**, **mental**, and **social** states of being Capacity to **perform** and **engage** in life in a way that is consistent with that **individual’s needs and wants**
Health Education [28]	Any combination of **learning experiences** designed to facilitate **voluntary adaptations** of behavior conducive to health
Health Promotion [28]	Any **combination** of health education and related organizational, economic, and environmental supports for behavior of individuals, groups, or communities conducive to health **Science** and **art** of helping people change their lifestyle to move toward a state of optimal health
Fitness [26]	Dynamic physical state—comprising cardiovascular/pulmonary endurance; muscle strength, power, endurance, and flexibility; relaxation; and body composition—that allows optimal and efficient performance of daily and leisure activities
Wellness [29,30]	Active process of becoming aware of and making choices toward a more successful existence Multidimensional ✓ **Emotional:** coping skills ✓ **Intellectual:** open-minded ✓ **Spiritual:** harmony; inner peace ✓ **Occupational:** enjoys role(s) in society ✓ **Social:** performs role(s) effectively, comfortably, and without harming others ✓ **Physical:** healthy body ✓ **Environmental:** pursuit of harmony with the surroundings and the world ✓ **Cultural:** awareness of, acceptance of, and appreciation for diverse cultures; accepting one’s own culture ✓ **Financial:** satisfaction with current and future financial situations
Prevention [17]	Activities that are directed toward ✓ Achieving and restoring optimal functional capacity ✓ Minimizing impairments, functional limitations, and disabilities ✓ Maintaining health (thereby preventing further deterioration or future illness) ✓ Creating appropriate environmental adaptations to enhance function Three levels of prevention: ✓ **Primary:** preventing a disease in a susceptible or potentially susceptible population through specific measures such as general health promotion efforts (e.g., assist patients/clients to stop smoking before they develop cardiovascular disease) ✓ **Secondary:** efforts to decrease the duration of illness, severity of disease, and sequelae through early diagnosis and prompt intervention (e.g., assist patients/clients to adopt a physical activity program to enhance their rehabilitation program and optimize recovery) ✓ **Tertiary:** efforts to limit the degree of disability and promote rehabilitation and restoration of function in patients/clients with chronic and irreversible diseases (e.g., encourage patients/clients to be more physically active to reduce stress and to slow down the progression of Parkinson’s disease)
Occupational Imbalance [31]	A lack of balance or disproportion of occupation resulting in decreased well-being
Deprivation [31]	Deprivation of occupational choice and diversity because of circumstances beyond the control of individuals or communities
Alienation [31]	Sense of isolation, powerlessness, frustration, loss of control, and estrangement from society or self as a result of engagement in occupation that does not satisfy inner needs

**Table 4:** Selected health communication strategies.

Strategy	Major Concepts
Motivational Interviewing	• Spirit – Collaboration – Evocation – Autonomy – Compassion • Principles – Expressing empathy – Developing discrepancy – Rolling with resistance – Supporting self-efficacy • Tools – Open-ended questions – Affirmations – Reflections – Summaries
5 A’s Counseling Approach to Lifestyle Change	Ask: Identify and document unhealthy lifestyle for every patient at every visit. Advise: In a clear, strong, and personalized manner, urge lifestyle change as needed. Assess: Is the patient/client willing to make a lifestyle change at this time? Assist: For the patient willing to make a lifestyle change, use counseling and strategies to help him or her successfully change. Arrange: Schedule follow-up contact, in person or by telephone, preferably within the first week after the commitment to change.
5 R’s Counseling Approach to Lifestyle Change	Relevance: Encourage the patient to indicate why lifestyle change is personally relevant. Risks: Ask the patient to identify potential negative consequences of an unhealthy lifestyle. Rewards: Ask the patient to identify potential benefits of lifestyle change. Roadblocks: Ask the patient to identify barriers or impediments to lifestyle change. Repetition: The motivational intervention should be repeated every time an unmotivated patient has an interaction with a clinician. Patients/clients who have failed in previous lifestyle change attempts should be told that most people make repeated attempts at lifestyle change before they are successful.

**Table 5:** Steps in the predisposing, reinforcing, and enabling constructs in educational/environmental diagnosis and evaluation (PRECEDE) policy, regulatory, and organizational constructs in educational and environmental development (PROCEED) model of health program planning [36].

Step	Description
Social Assessment	• Assess social and quality-of-life concerns of a population. • Relationship between health and quality of life is reciprocal. • Assure that planner is more likely to develop the most relevant program possible.
Epidemiological Assessment	• Identify specific health problems that are relevant to quality-of-life concerns. • Conduct secondary data analysis using existing data sources. • Explore global and local data sources. • Help set priorities and write program goals and objectives.
Behavioral and Environmental Diagnosis	• Identify factors internal and external to the individual that are causally linked to the occurrence and severity of the health problem. • Once listed: – Rank for importance. – Rank for changeability.
Educational and Ecological Assessment	• Examine factors that collectively influence the likelihood that behavioral and environmental change will occur. • **Predisposing factors:** antecedents to behavior that provide the rationale or motivation for the behavior. • **Reinforcing factors:** follow a behavior that provides for continuing reward or incentive for persistence or repetition of the behavior. • **Enabling factors:** antecedents to behavior that allow a motivation to be realized.
Administrative and Policy Assessment	• Identify policies, resources, and circumstances prevailing in the program’s organizational context that could facilitate or hinder program implementation.
Implementation	• Pilot-test proposed program. • Conduct formative evaluation—explore the execution of program elements and change immediately as needed.
Process Evaluation	• Determine the extent to which the program is implemented according to its protocol.
Impact Evaluation	• Examine short-term effects, including predisposing, reinforcing, and enabling factors as well as behavioral and environmental factors.
Outcome Evaluation	• Examine long-term effects, including health and quality-of-life indicators.
